# Segmentation-Free 3D Deep Learning for CT-Based Chronic Rhinosinusitis Classification

**DOI:** 10.3390/jimaging12100514

**Published:** 2026-10-15

**Authors:** Paolo Dalena, Martina Scagni, Ian Postuma, Francesca Brero, Alessandro Lascialfari, Johannes Haubold, Cornelius Deuschl, Stephan Lang, Stefan Mattheis, Kerstin Stähr

**Affiliations:** 1 Department of Otorhinolaryngology, Head and Neck Surgery, University Hospital Essen, University of Duisburg-Essen, Hufelandstraße 55, 45147 Essen, Germany; 2 Department of Physics, University of Pavia, Via Agostino Bassi 6, 27100 Pavia, Italy; 3 National Institute for Nuclear Physics (INFN), Pavia Division, Via Agostino Bassi, 6, 27100 Pavia, Italy; 4 Department of Diagnostic and Interventional Radiology and Neuroradiology, University Hospital Essen, University of Duisburg-Essen, Hufelandstraße 55, 45147 Essen, Germany

**Keywords:** chronic rhinosinusitis, computed tomography, deep learning, three-dimensional convolutional neural network, HiResCAM, paranasal sinuses

## Abstract

Chronic rhinosinusitis (CRS) is routinely assessed with computed tomography (CT), but CT-based differentiation between CRS with nasal polyps (CRSwNP) and without nasal polyps (CRSsNP) remains challenging. We retrospectively evaluated a segmentation-free three-dimensional convolutional neural network (3D CNN) for three-class classification using an audited cohort of 776 adult CT examinations: 155 CRSwNP, 222 CRSsNP, and 399 endocrine orbitopathy (EO) non-CRS controls. The volumes were standardized to 150 × 150 × 150 voxels. Baseline, augmentation, and augmentation-plus-L2 configurations were compared exclusively by validation macro-F1 before the primary locked-test evaluation of the selected compact model; post hoc architecture and sensitivity analyses were subsequently performed on the same frozen test set and were considered exploratory. On the 155-patient held-out test set, the validation-selected compact model achieved an accuracy of 0.748 (95% CI 0.684–0.813), balanced accuracy of 0.683 (0.602–0.763), macro-F1 of 0.691 (0.607–0.770), and macro one-versus-rest AUC of 0.882 (0.841–0.919). Performance was strongest for EO-control discrimination (one-versus-rest AUC 0.959), whereas direct CRSsNP-versus-CRSwNP discrimination was more limited (AUC 0.704; 95% CI 0.574–0.827). The CRSwNP recall was 0.452, and 15 of 31 CRSwNP cases were classified as CRSsNP. Thus, the aggregate macro-AUC was influenced predominantly by the stronger separation of EO controls from CRS and should not be interpreted as uniformly strong CRS phenotype discrimination. In a post hoc architecture sensitivity analysis, a 3D ResNet-10 achieved a higher observed test macro-F1 of 0.742, but used approximately 206 times as many parameters as the compact model. The HiResCAM maps were technically valid in all test cases, although 38/155 maps (24.5%) peaked outside an intensity-derived patient envelope that was not a sinus segmentation mask. These findings provide a research-stage proof of concept for future studies on CRS subtype discrimination; however, potential demographic- and acquisition-related shortcuts, together with the absence of external validation, preclude any claim of clinical readiness.

## 1. Introduction

Chronic rhinosinusitis (CRS) represents a substantial public health burden. Population-based studies estimate a prevalence of approximately 8% in the adult population, with an apparent increase in prevalence over the past 40 years [1]. For example, it accounts for millions of outpatient visits annually in the United States and Europe [2]. It is also associated with impaired quality of life, sleep disturbance, reduced work productivity, and considerable economic cost, with indirect costs related to lost productivity of more than ten billion dollars per year in the United States [1,2]. Patients also tend to report general health-related consequences, such as fatigue and impaired cognition, which have practical implications for how treatment success is defined and measured [2].

It is a relapsing inflammatory disease of the nasal and paranasal mucosa with nasal obstruction, facial pain or pressure, discharge, or impaired olfaction as typical symptoms, and is diagnosed when compatible symptoms persist for more than 12 weeks together with objective evidence of sinonasal inflammation [3,4]. It is further classified into CRS with nasal polyps (CRSwNP) and CRS without nasal polyps (CRSsNP). This distinction is clinically relevant because these phenotypes differ in their inflammatory biology, recurrence risk, medical responsiveness, and surgical planning [3,5]. CRSsNP is typically characterized by neutrophilic and fibrotic mucosal changes, whereas CRSwNP is frequently associated with type 2, eosinophil-driven inflammation, particularly in Western populations [5,6], although mixed and non-type 2 endotypes are increasingly recognized worldwide [6,7].

In addition to the economic impact, CRS phenotype classification masks a further substantial biological heterogeneity [6,7] that plays a relevant role. Endotype-driven classification based on the underlying inflammatory pathways (eosinophilic-versus-non-eosinophilic CRSwNP/type 2 versus non-type 2) has been proposed to better align the treatment response with the introduction of biologic therapies targeting specific cytokine pathways [5,6]. Endotyping currently requires biomarker assays or a detailed histopathological review, which are not uniformly available in routine clinical workflows and are impractical for large-scale or repeated assessment.

Deep learning models trained on sinus computed tomography (CT) have recently been used to predict such endotypes directly from imaging, achieving high discriminative performance and suggesting that volumetric CT features contain clinically meaningful information [8,9].

CT-based phenotype separation between CRSsNP and CRSwNP can be challenging even for experienced readers, because both processes present as soft-tissue attenuation within the sinonasal cavities, and their radiologic appearance can overlap substantially, particularly in recurrence or chronic cases [3,5]. The Lund–Mackay score remains the most widely used CT-based score system for CRS, but it was originally designed as a simple region-based opacification score rather than as a tool for phenotype differentiation [10,11]. It primarily quantifies regional opacification and does not directly measure the symptomatic burden. These limitations have motivated efforts to develop three-dimensional and reproducible CT-based scoring approaches, several of which now incorporate deep learning-based segmentation as a preprocessing step.

The objective of this study is to develop and internally evaluate a segmentation-free 3D CNN for CT-based classification of EO non-CRS controls, CRSsNP, and CRSwNP. In contrast to endotype prediction and volumetric disease burden scoring, the present work addresses separation of clinically used CRS phenotypes and controls. We use a frozen patient-level split, validation-only model selection, a post hoc reviewer-requested 3D ResNet-10 benchmark, sex-stratified and sex-matched sensitivity analyses, and a quantitative High-Resolution Class Activation Mapping (HiResCAM) audit. The attribution analysis measures the positive attribution within an intensity-derived patient envelope, at its border, and in the external air/background; it is designed to identify possible shortcut signals rather than to prove sinus-specific or causal model attention.

## 2. Related Work

### 2.1. Deep Learning in Medical Image Analysis

Deep learning has substantially improved automated medical image analysis over the past decade by enabling hierarchical feature learning directly from imaging data. The use of convolutional neural networks (CNNs) has demonstrated strong performance across lesion detection and organ segmentation, often matching expert-level performance in narrowly defined tasks. The extension of these architectures to three-dimensional medical imaging data, such as CT and Magnetic Resonance (MRI) volumes, has further enabled the direct exploitation of volumetric spatial context, which is particularly relevant for anatomically complex regions such as the paranasal sinuses, where the disease burden and anatomical variation are inherently three-dimensional [12,13].

### 2.2. Deep Learning in Sinonasal and Paranasal Sinus Imaging

In sinonasal imaging, prior work has addressed many relevant aspects of the pathology, which also have an impact on therapeutic decisions. Humphries et al. developed a CNN-based volumetric assessment of paranasal sinus opacification on CT, demonstrating how automated volumetric quantification can approximate expert-derived opacification scores [14].

Jeon et al. evaluated deep learning for the diagnosis of paranasal sinusitis using multi-view radiographs and reported competitive diagnostic performance [15].

Choi et al. instead proposed a deep learning-based fully automatic segmentation approach for the maxillary sinus on cone-beam CT images [16], while Whangbo et al. extended this approach to multi-class segmentation of the paranasal sinuses on conventional CT, reporting high segmentation accuracy in the presence of mucosal disease, although its performance was reduced in cases with severe inflammatory changes [17]. Complementary open-source segmentation pipelines have also been proposed to support reproducible, clinician-led paranasal sinus research without reliance on proprietary software [18].

### 2.3. Deep Learning for CRS Phenotyping and Endotyping

Deep learning on sinus CT to predict the eosinophilic-versus-non-eosinophilic endotype in CRSwNP has also shown strong discriminative performance and suggests that volumetric CT texture and distribution patterns carry endotype-relevant information [8].

A combined U-Net and DeepLabV3 approach was also tested by Hua et al. to distinguish eosinophilic from non-eosinophilic CRS on preoperative sinus CT, achieving area-under-the-curve (AUC) values in the range of 0.85–0.90 when predictions were aggregated at the patient level [9].

Semi-supervised three-dimensional volumetric CT scoring using deep learning-based semantic segmentation through the Lund–Mackay score demonstrated improved correlation with clinical parameters compared with traditional two-dimensional staging [19].

A recent systematic review of artificial intelligence-enhanced cone-beam CT for CRS reported high diagnostic accuracy in sinusitis detection, mucosal thickening quantification, anatomical variant identification, and CRS classification [20].

Complementary models have been proposed for sinusitis diagnosis on conventional CT, often achieving accuracy in the mid-80% range and outperforming human readers in specific settings, but they primarily focus on binary or regional sinusitis classification rather than comprehensive CRS phenotype separation [21].

### 2.4. Explainability in Deep Learning for Medical Imaging

Explainability is increasingly being recognized as critical for the clinical translation of deep learning models in medical imaging: high aggregate accuracy alone is insufficient if the underlying model relies on spurious acquisition artifacts, patient borders, or background regions rather than the clinically relevant anatomy [22]. Gradient-weighted Class Activation Mapping (Grad-CAM) has become one of the most widely used post hoc explainability techniques in medical imaging because it does not require architectural modification [23] and can be applied to any convolutional network with a differentiable classification head. It should also be noted that HiResCAM and related saliency-based methods do not always localize the diagnostically relevant regions in medical images, and that their outputs can vary substantially across architectures trained on the same task, underscoring the need for systematic, quantitative auditing of model attention rather than purely qualitative visual inspection [24].

### 2.5. Our Work

The preliminary findings related to this project were presented as a one-page German-language conference abstract in 2024, based on a subset of the retrospective cohort analyzed in the present study. The preliminary conference abstract should therefore not be considered independent evidence, replication, or external validation of the present findings. The current full-length study substantially extends that preliminary report by analyzing the complete audited cohort and providing a frozen patient-level development and internally held-out test design, validation-only model selection, duplicate-content auditing, subtype-specific performance estimates with confidence intervals, recorded-sex sensitivity analyses, a post hoc 3D ResNet-10 benchmark, and quantitative HiResCAM attribution auditing [25].

Taken together, the prior work in CRS imaging has predominantly addressed endotype prediction from CT or volumetric burden scoring and segmentation-based modification of traditional staging systems. Relatively less attention has been paid to segmentation-free three-class CT classification of EO non-CRS controls, CRSsNP, and CRSwNP, together with the explicit auditing of demographic sensitivity, architecture choice, and attribution outside patient anatomy [8,9,14,19,20,26].

The present study addresses this methodological gap using the complete audited cohort, a compact 3D CNN and a frozen validation-selection workflow. We additionally report the direct CRSsNP-versus-CRSwNP AUC, recorded-sex sensitivity analyses, a post hoc 3D ResNet-10 comparison, and positive HiResCAM attribution quantified relative to a technical patient-envelope mask. The latter is an exploratory quality-control analysis and not a validated measure of attention to the paranasal sinuses.

## 3. Materials and Methods

### 3.1. Patients and Data

This retrospective study used CT examinations from adults treated at the Department of Otorhinolaryngology, Head and Neck Surgery, University Hospital Essen, between 2015 and 2023. The scans were acquired by multiple radiologic providers across Germany and were available from the institutional clinical archive. The acquisition center identity, scanner model, reconstruction kernel, field of view, and other protocol metadata were not consistently retrievable. The cohort was therefore a retrospectively assembled multi-provider dataset, not an external or center-stratified validation cohort.

The final dataset included 776 CT examinations: 155 CRSwNP, 222 CRSsNP, and 399 controls with endocrine orbitopathy (EO) and no clinical or radiologic evidence of sinonasal inflammatory disease who served as non-CRS controls (Table 1). Absence of sinonasal disease in the control cohort was confirmed through independent radiological review of each CT examination by a board-certified radiologist, combined with the absence of documented sinonasal symptoms or ENT referral in the history. EO-related demographic and extra-sinonasal imaging characteristics may differ from those of both CRS cohorts and should therefore be considered when interpreting control-versus-CRS discrimination. Cases with unclear or incidental sinonasal findings on imaging were excluded from the control group. CRSwNP and CRSsNP labels were assigned using the routine multidisciplinary clinical reference standard, integrating symptom history, nasal endoscopy, and CT findings. All CRS cases underwent endoscopic sinus surgery and had a postoperative histopathological assessment. However, CRSsNP-versus-CRSwNP classification was based on the routine multidisciplinary clinical reference standard, including preoperative symptom history, nasal endoscopy, and CT findings; histopathology was used to confirm inflammatory sinonasal disease and exclude alternative pathology. Primary and recurrent cases were pooled within each CRS phenotype. The CRSsNP and CRSwNP labels represent clinically assigned phenotypes based primarily on the presence or absence of nasal polyps and should not be interpreted as inflammatory endotypes. Standardized endoscopic severity scores, asthma status, allergic phenotype, detailed prior-surgery status, systemic inflammatory markers, and tissue-level endotype biomarkers were not consistently available in a standardized format across the entire cohort and were therefore not incorporated as model inputs or adjustment variables. Exclusion criteria were age below 18 years, incomplete CT coverage of the paranasal sinuses, insufficient image quality, and sinonasal pathology unrelated to CRS. Pediatric examinations were excluded because the volumetric standardization differs substantially between pediatric and adult populations. During cohort assembly, 350 examinations were excluded because of age below 18 years, incomplete coverage of the paranasal sinuses, insufficient image quality, prior sinonasal conditions unrelated to CRS, unclear or incidental inflammatory findings in potential EO controls, duplicate examinations, or unavailable data required for definitive clinical–radiologic labeling.

### 3.2. CT Preprocessing

The acquisition parameters were heterogeneous. Each three-dimensional NIfTI image was loaded and checked for the finite voxel values. A bounding box was defined around voxels greater than −300 HU, and the historical exclusive upper-bound crop was reproduced. The cropped volume was resampled to isotropic 1 mm spacing and then cropped or padded to 150 × 150 × 150 voxels, using end alignment on one axis and centered alignment on the other two axes. The intensities were clipped to −1000 to 300 HU and linearly scaled to 0–1; out-of-field voxels were assigned the clipped air value. The audit verified the source and output checksums, source dimensions and spacing, finite values, and the final shape for every included examination. It did not contain anatomical landmark or sinus-segmentation quality control proving complete retention of the frontal, ethmoid, sphenoid, and maxillary sinuses in every patient. The residual variation in the scanner, reconstruction, field of view, and background characteristics could not be quantified from the available metadata.

### 3.3. Model Architecture

The primary model was a compact 3D CNN operating on 150 × 150 × 150 single-channel volumes. It comprised three 3 × 3 × 3 Conv3D layers with 16 filters and ReLU activation, two interleaved 4 × 4 × 4 max-pooling layers, a flattening operation, a 16-unit ReLU dense layer, and a three-unit softmax output. The model contained 69,667 trainable parameters. The compact design was chosen pragmatically to constrain memory demand and model capacity for 150^3^ inputs and a cohort of this size; it was not assumed to be architecturally optimal. The categorical cross-entropy was optimized with Adam using an initial learning rate of 10^−4^, β1 = 0.9, β2 = 0.999, ε = 10^−8^, and AMSGrad. A scheduler halved the learning rate after five epochs without improvement in the validation loss, to a minimum of 5 × 10^−6^.

As a post hoc architecture sensitivity analysis, a 3D ResNet-10 was trained on the identical frozen split. It used one 7 × 7 × 7 stem convolution; four residual stages with one block per stage and 64, 128, 256, and 512 filters; global average pooling; and a three-class output. It contained 14,362,819 parameters. The benchmark used the frozen split and the selected compact model’s augmentation, L2 coefficient, class weights, batch size, epoch budget, and minimum-validation-loss checkpoint rule. Because the compact model’s locked-test result already existed when this benchmark began, ResNet-10 was not eligible to alter primary model selection.

### 3.4. Training and Evaluation

A single patient-level split was frozen before training using seed 42 and stratification by class, primary/recurrent cohort, and recorded sex. The training, validation, and locked-test sets contained 466, 155, and 155 patients, respectively. Exact duplicate content was removed before splitting, and no patient identifier occurred in more than one partition. The split was not stratified by center, scanner, or protocol because those metadata were unavailable.

Three eligible compact-CNN configurations were trained on the same split for 300 epochs with an effective batch size of 1: baseline, augmentation, and augmentation plus L2 regularization. Augmentation comprised a random rotation from −15° to +15° and translations sampled from −10 to +10 voxels on two axes and 0 to 3 voxels on the third axis. The L2 coefficient was 0.001. No early stopping was used. Within each run, the checkpoint with minimum validation loss was retained. The eligible candidate with the highest validation macro-F1 was then selected; the locked test set was not used for checkpoint or candidate selection.

Balanced inverse-frequency class weights were computed from the training split as wc = N/(3nc), yielding 0.647 for EO controls, 1.168 for CRSsNP, and 1.670 for CRSwNP. An otherwise identical unweighted augmentation-plus-L2 model was trained as a validation-only sensitivity analysis and was ineligible for model selection.

Predictions were assigned by argmax of the three softmax probabilities; no post hoc decision threshold or probability-calibration procedure was applied. We report the accuracy; balanced accuracy; macro-F1; class-specific precision, recall and F1; class-wise one-versus-rest AUC; macro one-versus-rest AUC; and direct CRSsNP-versus-CRSwNP AUC. The principal locked-test 95% confidence intervals were estimated with 2000 class-stratified bootstrap resamples. The macro-AUC is an unweighted mean of three one-versus-rest discrimination problems and is not equivalent to direct CRS-subtype discrimination.

To assess the demographic shortcut risk, the locked-test performance was calculated separately for patients recorded as female and male. In addition, 1000 repeated binary EO-control-versus-any-CRS samples were matched within the recorded-sex strata. Each repetition included 28 female and 12 male patients per outcome group. The empirical 2.5th and 97.5th percentiles summarize the variation across repetitions. These exploratory analyses address only the recorded binary sex variable and cannot remove EO-, center-, scanner-, protocol-, anatomical-, or field-of-view-related confounding.

### 3.5. HiResCAM and Attention Quantification

The initial diagnostics showed that conventional gradient-weighted Class Activation Mapping (Grad-CAM) was unreliable for the selected architecture, whose convolutional stack is followed by flatten and dense layers. Spatial averaging of last-layer gradients produced cancellation: conventional Grad-CAM using the predicted-class logit at the last convolutional layer was non-degenerate in only 1/30 stratified diagnostic cases. HiResCAM retained the element-wise spatial gradient-feature interaction and was valid in all diagnostic configurations. The definitive analysis therefore used the positive component of HiResCAM for the predicted-class logit at the final convolutional layer (‘conv3d_2’) [12]. A map was technically valid when all values were finite and its positive component exceeded the prespecified non-degeneracy threshold; the pipeline required at least 95% valid maps.

Each map was independently normalized and upsampled to input resolution. An intensity-derived patient envelope was estimated using non-air thresholding, axial hole filling, and connected-component selection. The positive attribution mass was summarized within the patient envelope, tissue, internal air, external air/background, and inner/outer border compartments. The location of the maximum was also classified as inside or outside the patient envelope. This envelope is a technical quality-control mask, not a clinician-reviewed paranasal sinus segmentation; the attribution validity therefore does not demonstrate anatomically faithful, sinus-specific, or causal model attention.

## 4. Results

### 4.1. Classification Performance

The audited cohort contained 776 patients. The locked test set comprised 155 patients: 79 EO non-CRS controls, 45 CRSsNP, and 31 CRSwNP. Candidate selection used the validation data only. The validation macro-F1 was 0.705 for the baseline, 0.703 with augmentation, and 0.781 with augmentation plus L2; the augmentation-plus-L2 configuration was therefore selected. The unweighted sensitivity model achieved a validation macro-F1 of 0.754 and was not eligible for selection (Table 2).

**Figure 1:**
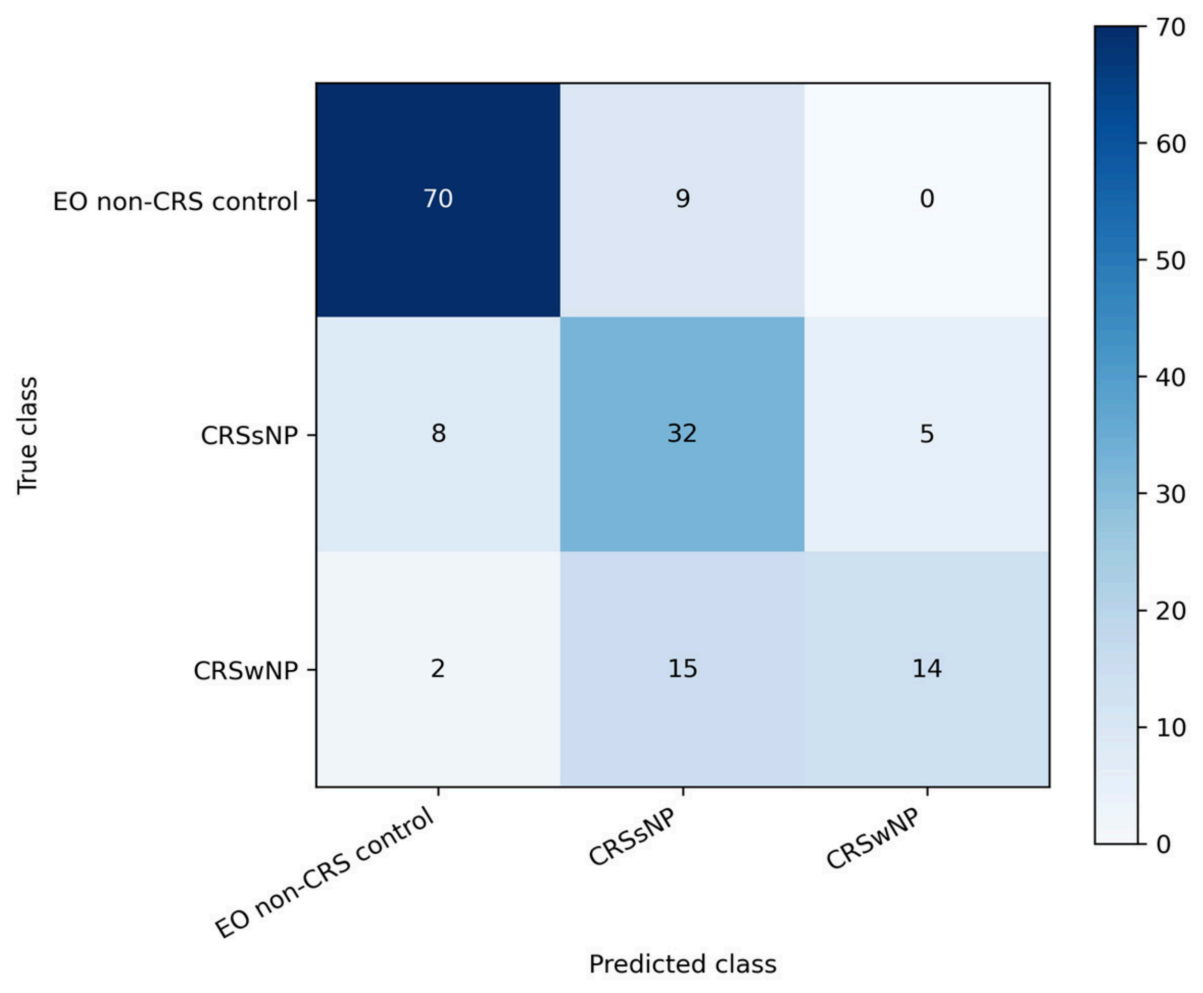
Locked-test confusion matrix for the validation-selected compact 3D CNN. The rows denote true classes and columns denote predicted classes (*n* = 155). The predominant disease-class error is prediction of CRSwNP as CRSsNP, which occurs in 15 of 31 CRSwNP cases.

On the locked test set, the selected compact model achieved accuracy of 0.748 (95% CI 0.684–0.813), balanced accuracy of 0.683 (0.602–0.763), macro-F1 of 0.691 (0.607–0.770), and macro one-versus-rest AUC of 0.882 (0.841–0.919). The direct CRSsNP-versus-CRSwNP AUC was 0.704 (0.574–0.827). The direct subtype AUC and CRSwNP recall of 0.452 show that clinically relevant subtype separation remained substantially weaker than EO-control-versus-CRS separation (Table 3). Of the 31 CRSwNP cases in the held-out test set, 15 were classified as CRSsNP, representing the predominant disease-class error (Figure 1).

Augmentation alone and augmentation plus L2 produced validation macro-AUC values of 0.904 and 0.907, respectively, while their validation macro-F1 values were 0.703 and 0.781. The AUC evaluated the score ranking across thresholds, whereas the macro-F1 was calculated from the argmax class predictions and was the prespecified candidate-selection metric. No calibration analysis was performed; the observed difference should therefore not be described as improved probability calibration.

Relative to the otherwise identical unweighted validation sensitivity model, balanced weighting increased the macro-F1 from 0.754 to 0.781 and CRSsNP recall from 0.636 to 0.795. The EO-control recall decreased from 0.963 to 0.938, and the CRSwNP recall decreased from 0.645 to 0.581. Weighting therefore improved the selection metric primarily through greater CRSsNP sensitivity rather than a uniform gain across minority classes.

### 4.2. Per-Class AUC Analysis

**Figure 2:**
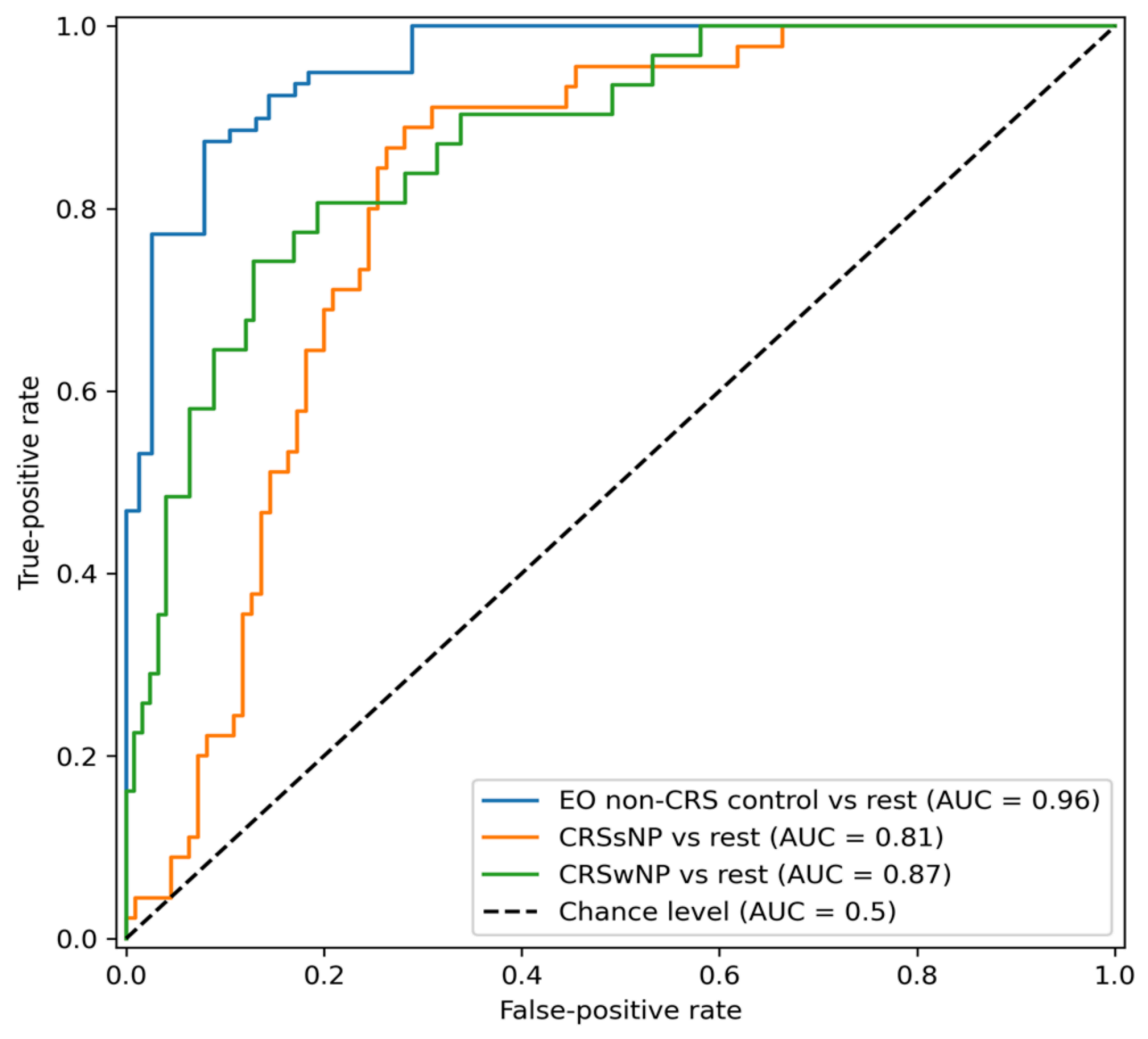
Locked-test one-versus-rest receiver operating characteristic curves for validation-selected compact 3D CNN. Macro-AUC is 0.882; because this measure includes comparatively strong EO-control-versus-CRS discrimination, it should not be interpreted as direct CRSsNP-versus-CRSwNP performance.

On the locked test set, the validation-selected augmentation-plus-L2 compact model achieved one-versus-rest AUCs of 0.959 for EO non-CRS controls, 0.814 for CRSsNP, and 0.873 for CRSwNP, yielding a macro one-versus-rest AUC of 0.882. The comparatively high EO-control AUC indicates that the aggregate macro-AUC was influenced by the stronger EO-control-versus-CRS separation. It should therefore not be interpreted as a direct measure of CRS subtype discrimination. The direct CRSsNP-versus-CRSwNP AUC was 0.704. The headline macro-AUC should primarily be interpreted as evidence of EO-control-versus-CRS separation rather than dependable discrimination between the two CRS phenotypes. Figure 2 shows the locked-test one-versus-rest ROC curves for the validation-selected compact model.

### 4.3. Architecture Sensitivity Analysis

The compact CNN contained 69,667 parameters, whereas the 3D ResNet-10 contained 14,362,819 parameters, a 206.2-fold difference. The compact CNN and ResNet-10 validation macro-F1 values were 0.781 and 0.760, respectively. On the same locked test set, ResNet-10 achieved accuracy of 0.806 (95% CI 0.748–0.865), balanced accuracy of 0.744 (0.666–0.821), macro-F1 of 0.742 (0.664–0.817), macro-AUC of 0.922 (0.888–0.952), and direct subtype AUC of 0.729 (0.603–0.838). The corresponding compact model values were 0.748, 0.683, 0.691, 0.882, and 0.704. The paired difference in the macro-AUC favored ResNet-10 by 0.040 (exploratory paired-bootstrap 95% CI 0.008–0.076), whereas the paired intervals for accuracy, balanced accuracy, macro-F1, and subtype AUC included zero. Because ResNet-10 was added after the primary locked-test result existed, this benchmark is descriptive and does not alter the prespecified validation-selected primary model (Table 4).

ResNet-10 was evaluated post hoc as an architecture benchmark to determine whether a standard 3D residual network would improve performance over the compact model. It achieved a higher observed test performance but required approximately 206 times more parameters. Because this comparison was introduced after completion of the prespecified compact model selection and locked-test evaluation, it should be interpreted as an exploratory architecture sensitivity analysis rather than as part of primary model selection. The bold type indicates the larger observed value. Because the architecture comparison was introduced post hoc, no confirmatory claim of statistical superiority is made, although the exploratory paired-bootstrap interval for the macro-AUC difference excluded zero.

### 4.4. Recorded-Sex Sensitivity Analysis

The locked test set included 95 patients recorded as female (67 EO controls, 17 CRSsNP, and 11 CRSwNP) and 60 recorded as male (12, 28, and 20, respectively). Among the recorded female patients, the accuracy was 0.800 (95% CI 0.726–0.863), balanced accuracy was 0.630 (0.516–0.746), macro-F1 was 0.637 (0.489–0.760), macro-AUC was 0.901 (0.847–0.946), and subtype AUC was 0.770 (0.556–0.941). Among the recorded male patients, the corresponding values were 0.667 (0.550–0.783), 0.671 (0.545–0.790), 0.670 (0.544–0.786), 0.811 (0.725–0.891), and 0.677 (0.502–0.841). The higher female accuracy was influenced by the much larger proportion of EO controls in that stratum; the balanced accuracy and macro-F1 did not show the same pattern. The female CRSwNP recall was 0.273 (3/11) and the male CRSwNP recall was 0.550 (11/20), with small denominators and wide uncertainty.

In 1000 repeated sex-matched binary EO-control-versus-any-CRS samples, each outcome group contained 28 recorded female and 12 recorded male patients. The mean accuracy and balanced accuracy were 0.859 (empirical 95% interval 0.825–0.888), and the mean AUC was 0.929 (0.911–0.946). This exploratory binary analysis addresses the recorded binary sex variable only. It does not establish equal three-class performance and cannot exclude EO-, site-, scanner-, reconstruction-, anatomical-, or field-of-view-related confounding (Table 5).

### 4.5. HiResCAM Attribution Findings

**Figure 3:**
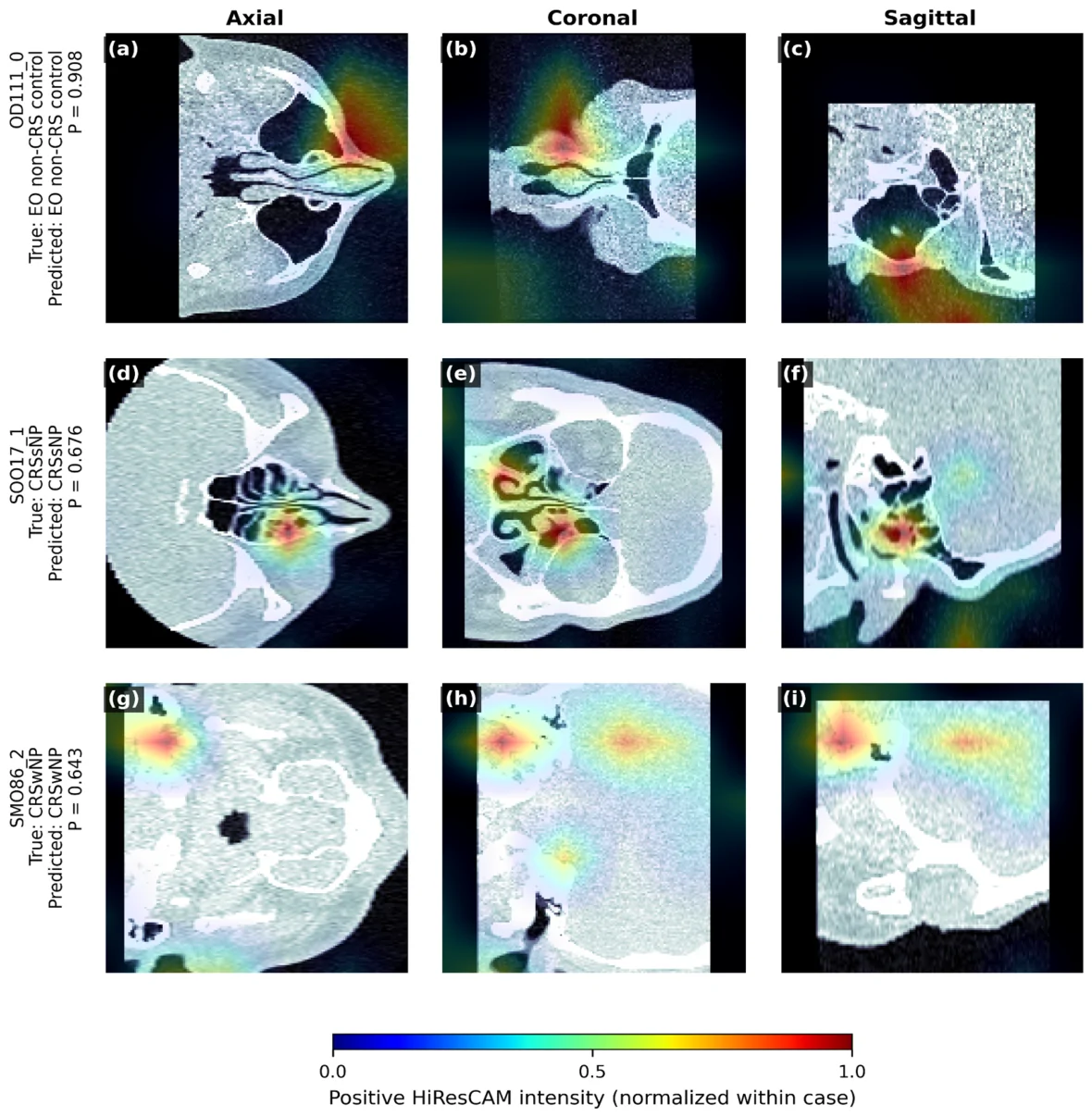
(**a**,**d**,**g**) Axial, (**b**,**e**,**h**) coronal, and (**c**,**f**,**i**) sagittal views. Positive HiResCAM attribution maps for representative correctly classified cases. Axial, coronal, and sagittal slices intersecting the maximum positive attribution are shown for one locked-test example from each true class: EO non-CRS control (OD111), CRSsNP (SOO17), and CRSwNP (SMO86). Maps are computed for the predicted-class logit at the last convolutional layer (‘conv3d_2’) and normalized independently within each case. Warmer colors indicate a stronger positive contribution to the predicted-class score. The examples demonstrate heterogeneous spatial attribution and should not be interpreted as proof of sinus-specific or causal model attention.

Positive HiResCAM maps targeting the predicted-class logit at ‘conv3d_2’ were finite and non-degenerate in all 155 locked-test examinations, exceeding the prespecified 95% validity requirement (Figure 3). Technical validity does not imply anatomically faithful localization. Across all cases, the mean positive attribution mass was 59.2% within the intensity-derived patient envelope (bootstrap 95% CI 57.1–61.3%), 40.8% in the external air/background (38.7–42.9%), and 23.2% in the combined inner/outer border band (22.5–23.8%). External-air attribution exceeded 50% in 41/155 cases (26.5%). The maximum was outside the patient envelope in 38/155 cases (24.5%; Wilson 95% CI 18.4–31.9%).

**Figure 4:**
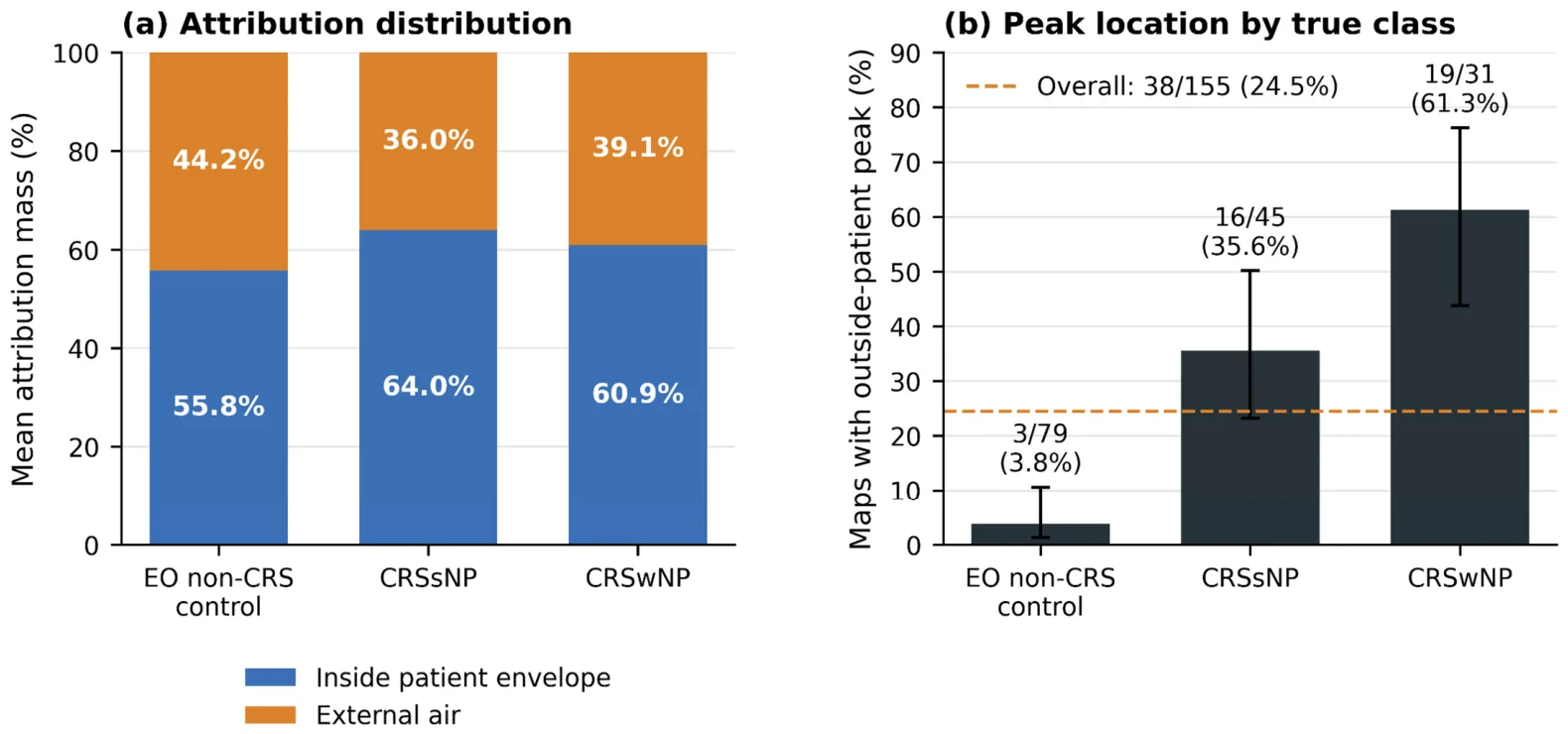
Quantitative localization of positive HiResCAM attribution in the locked test set. (**a**) Mean attribution mass inside the intensity-derived patient envelope and in external air, stratified by the true class. (**b**) Percentage of technically valid maps whose maximum is in the external air or outer border, with Wilson 95% confidence intervals. The dashed line shows the overall outside-patient peak rate (38/155, 24.5%). All 155 maps pass the prespecified finite, non-degenerate validity criterion. The patient envelope is an automated technical quality-control mask and not a clinician-reviewed sinus segmentation.

**Figure 5:**
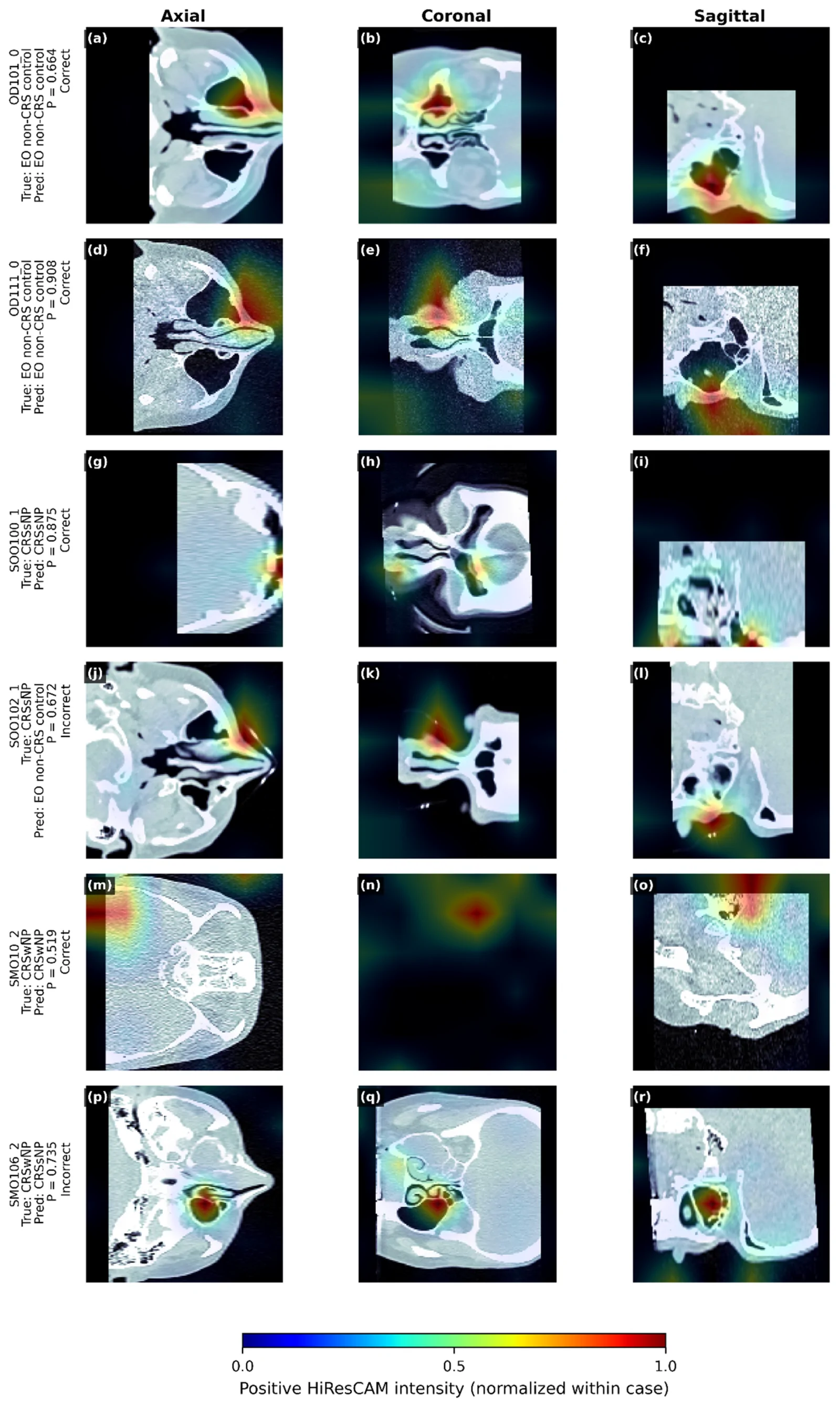
Positive HiResCAM peak slices for all six saved diagnostic examples. (**a**,**d**,**g**,**j**,**m**,**p**) Axial, (**b**,**e**,**h**,**k**,**n**,**q**) coronal, and (**c**,**f**,**i**,**l**,**o**,**r**) sagittal views. Two examples from each true class are shown, including correct and incorrect predictions. For each case, axial, coronal, and sagittal slices intersecting the maximum positive attribution are displayed. The maps target the predicted-class logit at ‘conv3d_2’ and are normalized independently within each case. These maps provide technical attribution diagnostics, not evidence of anatomically faithful or causal attention.

By the true class, outside-envelope peaks occurred in 3/79 EO controls (3.8%; Wilson 95% CI 1.3–10.6%), 16/45 CRSsNP cases (35.6%; 23.2–50.2%), and 19/31 CRSwNP cases (61.3%; 43.8–76.3%). The mean patient-envelope/external-air/border attribution fractions were 0.558/0.442/0.208 for the EO controls, 0.640/0.360/0.255 for CRSsNP, and 0.609/0.391/0.259 for CRSwNP (Table 6; Figure 4). The relatively frequent external and boundary emphasis, particularly among the CRS cases, is compatible with the field-of-view, acquisition, anatomy, or other shortcut effects (Figure 5). Because the envelope was not a sinus segmentation, these results do not establish whether the network used clinically relevant sinonasal structures.

## 5. Discussion

### 5.1. Principal Findings

In this retrospective internal evaluation, the validation-selected compact 3D CNN achieved locked-test accuracy of 0.748, macro-F1 of 0.691, and macro one-versus-rest AUC of 0.882. Performance was not uniform across the study groups. The strongest result was observed for EO-control discrimination (AUC 0.959), whereas direct CRSsNP-versus-CRSwNP discrimination was more limited (AUC 0.704; 95% CI 0.574–0.827). The CRSwNP recall was 0.452, and 15 of 31 CRSwNP cases were classified as CRSsNP. The aggregate macro-AUC was therefore driven predominantly by the comparatively easier EO-control-versus-CRS distinction and does not demonstrate dependable CRS phenotype discrimination. The principal finding is technical feasibility in a research setting rather than clinical readiness or validated CRS phenotyping.

The compact model was chosen pragmatically to constrain the parameter count and computation for 150^3^ inputs. The post hoc ResNet-10 benchmark demonstrated a resource–performance trade-off rather than compact model optimality. The compact model was approximately 206 times smaller and had a higher validation macro-F1, whereas ResNet-10 achieved better observed locked-test metrics. As the benchmark was initiated after the primary locked-test result existed, it cannot retrospectively participate in primary model selection. Prespecified multi-architecture comparisons in an independent external cohort are needed.

The corrected HiResCAM analysis produced technically valid maps in every locked-test case, but substantial attributions to the external air and at patient boundaries remained, with 38/155 maps peaking outside the patient envelope. These findings preclude a claim that the model consistently focuses on clinically relevant sinus anatomy. HiResCAM is therefore used as an exploratory technical audit and failure-analysis tool. Validation against clinician-reviewed sinus masks and targeted orbit-masked and field-of-view-matched ablations are required before making anatomical attention claims.

### 5.2. Clinical Implications

The present findings should be interpreted as a research-stage proof of concept. The internal test set does not establish performance in a healthy control population, across unseen centers or scanners, or in prospective clinical workflows. If subsequently confirmed in center-held-out external cohorts, the model could be studied as an adjunct to (not a replacement for) clinical assessment, nasal endoscopy, radiologist CT interpretation, histopathology where indicated, and multidisciplinary decision-making. Prospective reader studies should compare unaided and AI-assisted interpretation using diagnostic accuracy, inter-reader agreement, reporting time, confidence, and management impact.

### 5.3. Limitations

This study has several limitations. First, it is retrospective and lacks strictly external or center-held-out validation. Although the scans originated from multiple providers, the center, scanner, reconstruction, and protocol metadata were incomplete, and patients from the same provider may have been present across the development and test partitions. Patient-level separation and duplicate-content removal prevent direct patient and exact-image overlap, but they do not prevent shared provider, scanner, reconstruction, or workflow signatures across the partitions. Consequently, the held-out test set is internally independent at the patient level but is not an independent center-held-out cohort, and the results do not establish transportability to unseen institutions, scanners, referral populations, or acquisition protocols. Preprocessing cannot guarantee removal of acquisition signatures, and the external/border HiResCAM signal increases concern about field-of-view or acquisition shortcuts.

Second, the control group comprised patients with endocrine orbitopathy rather than neutral healthy volunteers and had a markedly different recorded-sex distribution: 84.7% female in the EO controls versus 38.7% in CRSsNP and 35.5% in CRSwNP. The sex-stratified and repeated sex-matched analyses address one measured characteristic but do not remove the EO-related, demographic, center, scanner, reconstruction, anatomical, or field-of-view confounding. The sex-matched analysis is binary and cannot establish equal three-class performance.

Third, the reconstruction reproduced the historical intensity-bounding-box crop, but no anatomical landmark or segmentation audit proved that every peripheral sinus was fully retained in every scan. Fourth, standardized endoscopic scores, asthma status, allergic phenotype, detailed prior-surgery information, systemic inflammatory markers, and systematic tissue-level biomarker or molecular-endotype data were not consistently available across the cohort. The reference labels therefore represent routine clinical CRSsNP and CRSwNP phenotypes rather than biologically validated inflammatory endotypes. The study cannot determine whether the model captures polyp status, inflammatory biology, disease burden, previous treatment or surgery, or other correlated imaging and clinical characteristics. Fifth, the reference labels are clinical phenotypes and no voxel-level sinus annotation was available. The intensity-derived patient envelope is not a sinus mask; HiResCAM cannot establish causal or sinus-specific model reasoning. Sixth, four cross-class duplicate pairs had unresolved labels. Excluding all eight affected images was conservative but does not replace clinical adjudication. Finally, the ResNet-10 benchmark and paired model comparison were post hoc and require confirmation in a prespecified external study.

### 5.4. Future Work

The required next step is a prespecified multicenter external-validation study in which at least one institution is held out in its entirety from model development. Before data transfer, the compact model, preprocessing pipeline, intensity window, resampling method, crop dimensions, normalization procedure, and decision rule should be frozen. The external cohort should preferably include consecutive adult patients and should record the acquisition center, scanner manufacturer and model, reconstruction kernel, slice thickness, field of view, contrast status, provider and referral pathway, prior sinus surgery, asthma and allergy status, nasal endoscopic findings, standardized endoscopic scores, and histopathological or biomarker data where available. The principal clinically relevant external endpoint should be direct CRSsNP-versus-CRSwNP discrimination, with the AUC, CRSwNP sensitivity, specificity, calibration, and confidence intervals prespecified. Control-versus-CRS discrimination should be evaluated as a separate endpoint using a clinically representative non-CRS control population; EO controls, if included, should be analyzed and reported separately. The external-study sample size should be determined a priori to estimate the direct subtype AUC and CRSwNP sensitivity with prespecified confidence interval precision.

Additional priorities are: (1) prespecified scanner- and protocol-level sensitivity analyses; (2) comparison with radiologists and otolaryngologists in unaided and AI-assisted workflows; (3) anatomical coverage quality control; (4) attribution validation against clinician-reviewed sinus segmentations; (5) orbit-masked and field-of-view-matched ablations; (6) prespecified comparison of compact and standard 3D architectures; (7) probability calibration and uncertainty analyses; and (8) prospective integration of standardized endoscopy, prior surgery, allergy/asthma status, histopathology, and tissue biomarkers for endotype-aware modeling.

## 6. Conclusions

In this retrospective internal evaluation, a compact segmentation-free 3D CNN separated EO non-CRS controls from CRS more reliably than it distinguished CRSsNP from CRSwNP. The validation-selected compact model achieved a macro one-versus-rest AUC of 0.882, but this aggregate result was influenced predominantly by EO-control discrimination (AUC 0.959). The direct CRSsNP-versus-CRSwNP AUC was 0.704, the CRSwNP recall was 0.452, and 15 of 31 CRSwNP cases were classified as CRSsNP. The results therefore demonstrate technical feasibility but not dependable CRS phenotyping. A post hoc 3D ResNet-10 achieved higher observed test metrics at approximately 206 times the parameter count, illustrating an exploratory resource–performance trade-off rather than architectural superiority. HiResCAM provided a coarse attribution audit but did not establish sinus-specific or causal model reasoning. Potential EO-related, demographic, provider, scanner, acquisition, anatomical, and field-of-view confounding cannot be excluded. Independent center-held-out multicenter validation, a richer clinical ground truth, and anatomically grounded attribution analyses are required before clinical utility, transportability, or deployment can be considered.

## Figures and Tables

**Table 1:** Patient groups and demographics.

Group	CT Scans	Male, *n* (%)	Female, *n* (%)	Mean Age	Age SD
CRSwNP ^1^	155	100 (64.5)	55 (35.5)	54.0	14.6
CRSsNP ^2^	222	136 (61.3)	86 (38.7)	54.8	14.6
Control ^3^	399	61 (15.3)	338 (84.7)	51.2	11.8
Total	776	297 (38.3)	479 (61.7)	52.8	13.3

^1^ CRSwNP = chronic rhinosinusitis with nasal polyps; ^2^ CRSsNP = chronic rhinosinusitis without nasal polyps. ^3^ Controls are patients with EO and no sinonasal inflammatory disease.

**Table 2:** Validation-only compact model selection and weighting sensitivity analysis.

Candidate	Augmentation	L2	Class Weights	Eligible	Validation Macro-F1
Baseline	No	0	Balanced	Yes	0.705
Augmentation	Yes	0	Balanced	Yes	0.703
Augmentation + L2	Yes	0.001	Balanced	Yes	0.781
Augmentation + L2, unweighted	Yes	0.001	None	No; sensitivity only	0.754

**Table 3:** Locked-test performance of the validation-selected compact 3D CNN.

Class	*n*	Precision	Recall	F1	One-Versus-Rest AUC
EO non-CRS control	79	0.875	0.886	0.881	0.959
CRSsNP	45	0.571	0.711	0.634	0.814
CRSwNP	31	0.737	0.452	0.560	0.873

**Table 4:** Descriptive post hoc architecture sensitivity analysis on the identical frozen split. The comparison is not prospectively specified and does not establish architectural superiority or optimality.

Architecture	Parameters	Validation Macro-F1	Test Accuracy	Test Balanced Accuracy	Test Macro-F1	Test Macro-AUC	Test Subtype AUC
Compact 3D CNN	69,667	0.781	0.748	0.683	0.691	0.882	0.704
3D ResNet-10	14,362,819	0.760	0.806	0.744	0.742	0.922	0.729

**Table 5:** Exploratory locked-test performance stratified by recorded sex.

Recorded Sex	*n*	Class Composition (EO/CRSsNP/CRSwNP)	Accuracy	Balanced Accuracy	Macro-F1	Macro-AUC	Subtype AUC
Female	95	67/17/11	0.800	0.630	0.637	0.901	0.770
Male	60	12/28/20	0.667	0.671	0.670	0.811	0.677

**Table 6:** Positive HiResCAM attribution by the true class in the locked test set. The mass fractions are descriptive means; the border band overlaps the inside/outside partition and is not an additional mutually exclusive mass.

True Class	*n*	Patient-Envelope Mass	External-Air Mass	Border Mass	Outside-Envelope Peak, *n* (%)
EO non-CRS control	79	0.558	0.442	0.208	3 (3.8)
CRSsNP	45	0.640	0.360	0.255	16 (35.6)
CRSwNP	31	0.609	0.391	0.259	19 (61.3)
Overall	155	0.592	0.408	0.232	38 (24.5)

## Data Availability

The original contributions presented in this study are included in the article. Further inquiries can be directed to the corresponding author.

## Abbreviations

The following abbreviations are used in this manuscript:

3D CNN	three-dimensional convolutional neural network
AUC	area under the curve
CNNs	convolutional neural networks
CRS	chronic rhinosinusitis
CRSsNP	chronic rhinosinusitis without nasal polyps
CRSwNP	chronic rhinosinusitis with nasal polyps
CT	computed tomography
EO	endocrine orbitopathy
Grad-CAM	gradient-weighted class activation mapping
HiResCAM	high-resolution class activation mapping
MRI	magnetic resonance
